# An Update on the Roles of circRNA-ZFR in Human Malignant Tumors

**DOI:** 10.3389/fcell.2021.806181

**Published:** 2022-02-02

**Authors:** Lang Liu, Haicun Wang, Shaobo Yu, Xin Gao, Guanglin Liu, Dongsheng Sun, Xingming Jiang

**Affiliations:** Department of General Surgery, The 2nd Affiliated Hospital of Harbin Medical University, Harbin, China

**Keywords:** circular RNA, circRNA-ZFR, malignant tumors, expression, mechanism

## Abstract

CircRNAs (circular RNAs) are single-stranded RNAs that form covalently closed loops and function as important regulatory elements of the genome through multiple mechanisms. Increasing evidence had indicated that circRNAs, which might serve as either oncogenes or tumor suppressors, played vital roles in the pathophysiology of human diseases, especially in tumorigenesis and progression. CircRNA-ZFR (circular RNA zinc finger RNA binding protein) is a circular RNA that had attracted much attention in recent years. It has been found that circRNA-ZFR was abnormally expressed in a variety of malignant tumors, and its dysregulated expression was closely related to tumor stage, cancer metastasis and patients’ prognosis. Recent studies had shown that aberrantly expressed circRNA-ZFR could regulate the malignant biological behaviors of tumors through various mechanisms; further exploration of circRNA-ZFR expression in tumors and its regulation on malignant biological behaviors such as tumor proliferation, invasion and drug resistance will provide new ideas for clinical tumors diagnosis and treatment.

## Introduction

The genome-wide studies showed that greater than 70% of the human genome is transcribed into RNAs, while only approximately 2% of the sequences have the capacity to encode proteins. The non-coding RNAs (ncRNAs), which were long assumed to be transcriptional noise, comprise the most proportion of the transcripts Covalently closed circRNAs (circular RNAs) were originally identified in plant viroids, yeast mitochondrial RNAs, and hepatitis virus (Kristensen et al., 2019; Xu et al., 2020). CircRNAs are a large class of endogenously expressed non-coding RNAs characterized by covalently closed loop structures with neither 5′ to 3′ polarity nor polyadenylated tail. Compared with linear RNAs, circRNAs with closed loop structure are very stable and resistant to RNase, and also highly specific in human tissues and cell samples (Patop et al., 2019; Yu et al., 2019; Chen et al., 2019). With deepening research, more and more circRNAs have been found to show abnormal expression in a variety of malignant tumors, and affected the tumor occurrence and development by targeting key genes (Huang et al., 2020; Lei et al., 2020; Zhou C. et al., 2020). CircRNAs dysregulation could promote proliferation, invasion and metastasis of tumor cells and inhibit cellular senescence and apoptosis by multiple mechanisms, including working as miRNA sponges, protein scaffolds, regulatory signals or transcript decoys. Additionally, circRNAs might serve as potential therapeutic targets and biomarkers for patients’ diagnosis or prognosis due to elevated stability, high efficiency and tissue specificity (Han et al., 2018; Zhang et al., 2018; Li et al., 2019).

**FIGURE 1:**
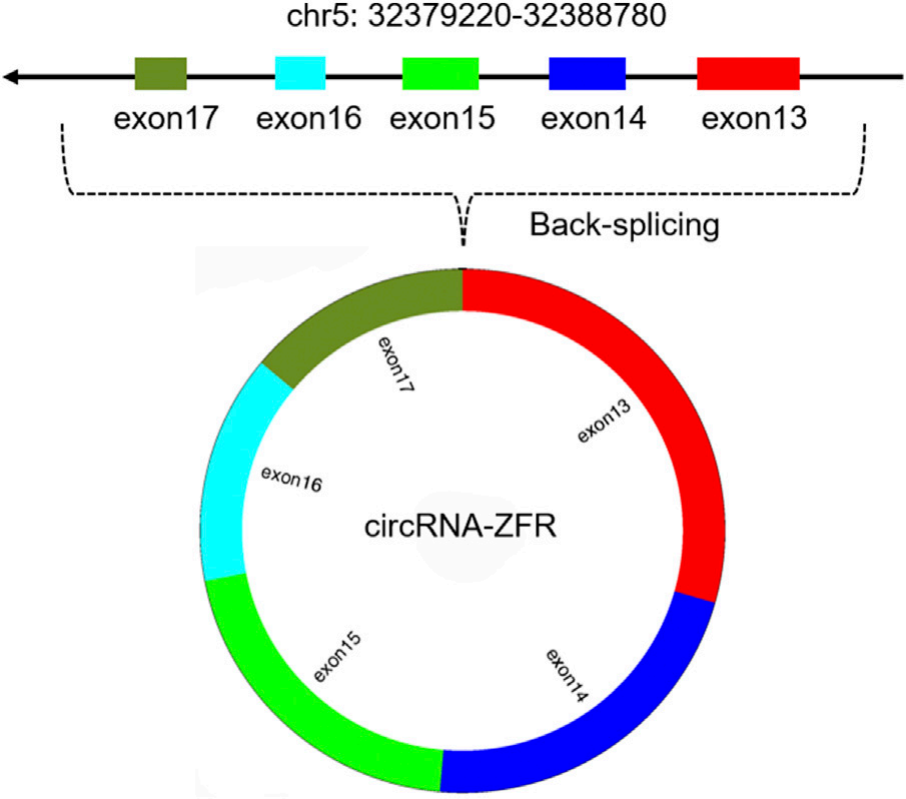
The chromosomic location and biogenesis of circRNA-ZFR.

**FIGURE 2:**
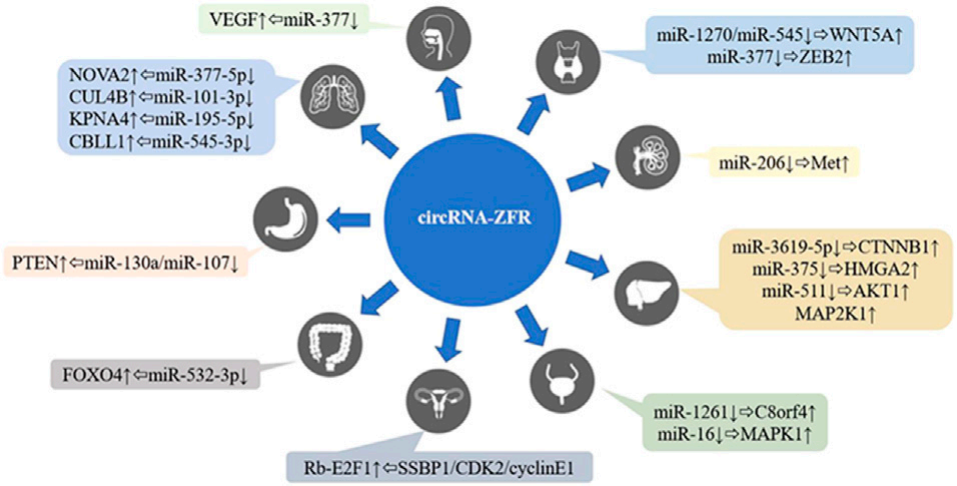
The regulatory network of circRNA-ZFR on human malignant tumors. HIF1A, hypoxia-inducible factor 1*α*; C8orf4, transcriptional and immune response regulator; MAPK1, mitogen-activated protein kinase 1; Met, met proto-oncogene; WNT5A, Wnt family member 5A; ZEB2, zinc finger E-box binding homeobox 2; E2F1, E2F transcription factor 1; VEGF, vascular endothelial growth factor; HMGA2, high mobility group AT-hook 2; CTNNB1, catenin beta 1; AKT1, AKT serine/threonine kinase 1; MAP2K1, mitogen-activated protein kinase kinase 1; NOVA2, NOVA alternative splicing regulator 2; CUL4B, cullin 4B; KPNA4, karyopherin subunit alpha 4; CBLL1, Cbl proto-oncogene like 1; PTEN, phosphatase and tensin homolog; FOXO4, forkhead box O4.

CircRNA-ZFR (zinc finger RNA binding protein) is a new circRNA (circBase ID: hsa_circ_0072088), which had been confirmed to be located on human chromosome 5p13.3 (Shown in Figure 1). As a member of the circular RNA family, circRNA-ZFR had been shown to be abnormally expressed in human malignant tumors such as hepatocellular carcinoma, breast cancer and thyroid carcinoma, and several researches also proved that circRNA-ZFR could impact the tumors development through affecting the glycolysis of tumor cells, regulating the tumor microenvironment and cell cycle (Li et al., 2020; Zhang et al., 2020). The clinical data analysis indicated that circRNA-ZFR expression was closely related to tumor TNM stage and patients’ prognosis. Current studies had shown that circRNA ZFR played roles as a tumor suppressor or oncogene in many tumors (Shown in Table 1); the regulatory mechanisms included acting as molecular sponges to competitively adsorb miRNA and then regulate the expression of target genes, or interacting with target RNA binding protein through specific RNA binding domain to form RNA protein complex, so as to affect the process of human malignant tumors (Shown in Figure 2).

**TABLE 1:** The expressions and regulation mechanisms of circRNA-ZFR on malignant tumors.

Cancer types	Expression	Related genes and pathways	Biological significance *in vitro*	Biological significance *in vivo*
Breast cancer	Upregulated	miR-758/HIF1A	Proliferation ↑, migration ↑, invasion ↑, glycolysis ↑, apoptosis ↓	Promote tumor growth
Thyroid carcinoma	Upregulated	miR-1261/C8orf4	Proliferation ↑, migration ↑ and invasion ↑	/
		miR-16/MAPK1	Proliferation ↑, migration ↑, invasion ↑, apoptosis ↓	/
Renal cell cancer	Upregulated	miR-206/Met	Proliferation ↑, migration ↑, invasion ↑, apoptosis ↓	/
Bladder cancer	Upregulated	miR-1270/miR-545/WNT5A	Proliferation ↑, migration ↑ and invasion ↑	Promote tumor growth
		miR-377/ZEB2	Proliferation ↑, migration ↑, invasion ↑, apoptosis ↓	/
Cervical cancer	Upregulated	Rb-E2F1	Proliferation ↑, migration ↑, invasion ↑	Promote tumor growth
Esophageal squamous cell carcinoma	Upregulated	miR-377/VEGF	Proliferation ↑, migration ↑, invasion ↑	Promote tumor growth
Hepatocellular carcinoma	Upregulated	miR-375/HMGA2	Proliferation ↑, migration ↑, invasion ↑, glycolysis ↑, apoptosis ↓	Promote tumor growth
		miR-3619-5p/CTNNB1	Proliferation ↑	/
		miR-511/AKT1	Proliferation ↑, migration ↑, invasion ↑ and apoptosis ↓	Promote tumor growth
		MAP2K1	Proliferation ↑	/
Non-small cell lung carcinoma	Upregulated	miR-377-5p/NOVA2	Proliferation ↑, migration ↑, invasion ↑ and apoptosis ↓	Promote tumor growth
		miR-101-3p/CUL4B	Proliferation ↑, migration ↑ and invasion ↑	/
		miR-195-5p/KPNA4	Proliferation ↑, migration ↑, invasion ↑, apoptosis ↓ and chemoresistance ↑	Promote tumor growth
		miR-545-3p/CBLL1	Proliferation ↑, migration ↑, invasion ↑, apoptosis ↓ and chemoresistance ↑	Promote tumor growth
Gastric cancer	Downregulated	miR-130a/miR-107/PTEN	Proliferation ↓ and apoptosis ↑	Curb tumor growth
Colorectal cancer	Downregulated	miR-532-3p/FOXO4	Proliferation ↓, migration ↓ and invasion ↓	/

HIF1A, hypoxia-inducible factor 1α; C8orf4, transcriptional and immune response regulator; MAPK1, mitogen-activated protein kinase 1; Met, met proto-oncogene; WNT5A, Wnt family member 5A; ZEB2, zinc finger E-box binding homeobox 2; E2F1, E2F transcription factor 1; VEGF, vascular endothelial growth factor; HMGA2, high mobility group AT-hook 2; CTNNB1, catenin beta 1; AKT1, AKT, serine/threonine kinase 1; MAP2K1, mitogen-activated protein kinase kinase 1; NOVA2, NOVA, alternative splicing regulator 2; CUL4B, cullin 4B; KPNA4, karyopherin subunit alpha 4; CBLL1, Cbl proto-oncogene like 1; PTEN, phosphatase and tensin homolog; FOXO4, forkhead box O4.

## Circular RNA Zinc Finger RNA Binding Protein in Malignant Tumors

### Circular RNA Zinc Finger RNA Binding Protein in Breast Cancer

Breast cancer (BC) is one of the most common gynecological cancers. The deteriorating environment and lifestyle flaws are raising the frequency of this cancer (Ellis and Ma 2019; Liang et al., 2019). The current view is that breast cancer is a stem cell disease characterized by the existence of cancer cells with stem-like features and tumor-initiating potential. Existing therapies were not universally-effective to this stem cell disease, and usually caused side effects, relapses, and high mortality rate (Dittmer 2018; Rossi et al., 2019). Therefore, it is necessary to seek ideal plans for the palliative treatment of advanced BC.

Chen et al. (2020) initially demonstrated that circRNA-ZFR expression was significantly increased in 70 BC tissues and tumor cells compared to the corresponding tissues. Moreover, circRNA-ZFR overexpression was remarkably correlated with tumor size, depth of invasion and TNM stage; Kaplan-Meier survival curves showed that the patients with low circRNA-ZFR expression had longer survival time. Functionally, downregulation of circRNA-ZFR significantly inhibited cell viability, migration and invasion and strongly promoted apoptosis; circRNA-ZFR silencing resulted in decreased glucose uptake, lactate product and ATP level. In xenograft model assays, tumor growth was remarkably stunted after transfecting low circRNA-ZFR expressed tumor cells. To further understand the roles of circRNA-ZFR in BC development, researchers performed detailed analysis for its targeted miRNAs and the results verified that circRNA-ZFR directly interacted with miR-578. In BC tissues and cell lines, miR-578 expression was significantly decreased and it was inversely correlated with circRNA-ZFR expression; and the miR-578 expression decreasing partially reversed the promoting effect of circRNA-ZFR on the tumor cells malignant biological behaviors. Furthermore, the researchers identified HIF1A (hypoxia-inducible factor 1*α*) as a functional target of miR-578 in regulating BC cell viability, migration, invasion, glycolysis and anti-apoptosis. In BC tissues and cell lines, HIF1A mRNA expression was positively correlated with circRNA-ZFR expression and negatively correlated with miR-578 expression. The results of luciferase report assay and RNA pull-down indicated that circRNA-ZFR modulated HIF1A expression via acting as a sponge of miR-578. These findings suggested that the aberrant expression of circRNA-ZFR in breast cancer promoted the tumor proliferation, invasion, migration and inhibited cell apoptosis by regulating miR-578/HIF1A axis.

## Circular RNA Zinc Finger RNA Binding Protein in Thyroid Carcinoma

By analyzing the Gene Expression Omnibus (GEO) dataset, Xiong et al. (2021) confirmed that circRNA-ZFR expression in TC (thyroid carcinoma) tissues was significantly upregulated compared with that in adjacent normal tissues; exogenously downregulated circRNA-ZFR expression suppressed the malignant behaviors of TC tumor cells. Mechanically, there was a complementary sequence in circRNA-ZFR for miR-16, and miR-16 could target the 3′-untranslated region of MAPK1 (mitogen-activated protein kinase 1); as a member of the MAPK family, MAPK1 is well known as an oncogene that is activated or highly expressed in various types of human cancer (Jiang et al., 2019; Deng et al., 2020; Janardhan et al., 2020). The western blot analysis results further demonstrated that augmented levels of circRNA-ZFR promoted MAPK1 expression in TPC-1 cells, while miR-16 augmentation partially counteracted the increased MAPK1 expression induced by circRNA-ZFR overexpression. In addition, the inhibition of cell viability, invasion and apoptosis induced by circRNA-ZFR knockdown were partially reversed in TPC-1 and IHH-4 cells after miR-16 depletion or MAPK1 promotion. All these data provided the evidence that circRNA-ZFR/miR-16/MAPK1 axis might serve as promotion effect for TC progression.

Wei et al. (2018) analyzed circRNA-ZFR expression in 41 pairs of PTC (papillary thyroid carcinoma) tissues and adjacent normal tissues. The results confirmed that circRNA-ZFR was remarkably overexpressed in PTC tissue specimens compared with that in paired non-neoplastic specimens, and increased circRNA-ZFR was significantly associated with tumor volume, depth of tumor invasion, lymph node metastasis, haematogenous metastasis, and patients’ poorer outcomes. Moreover, Kaplan-Meier curve analysis data indicated higher circRNA-ZFR expression in PTC patients was associated with worse prognosis. Functional experiments results illustrated that circRNA-ZFR knockdown significantly inhibited the proliferation, migration and invasion potential in TPC-1 and SW579 cells. By bioinformatics prediction and luciferase analysis, the researchers found that circRNA-ZFR could directly interact with miR-1261, and miR-1261 could target downstream oncogene C8orf4 (transcriptional and immune response regulator); Western blot and qRT-PCR results also verified that circRNA-ZFR knockdown and miR-1261 overexpression both significantly downregulated C8orf4 expression, while circRNA-ZFR overexpression abrogated the inhibition effect of miR-1261 mimics on C8orf4 expression. The rescue experiment results showed C8orf4 overexpression also attenuated the proliferation, migration and invasion-promoting effects of circRNA-ZFR silencing on PTC cells. Taken together, circRNA-ZFR exerted oncogenic roles via regulating miR-1261/C8orf4 axis in papillary thyroid carcinoma, which suggested circRNA-ZFR might be a potential therapeutic target.

## Circular RNA Zinc Finger RNA Binding Protein in Renal Cell Cancer

The qRT-PCR detection results showed that circRNA-ZFR expression was remarkably escalated in RCC (renal cell cancer) tissues as compared with para-carcinoma tissues, and circRNA-ZFR expression was also notably raised in CAKI-1, ACHN, A498 and KTCTL-26 cells. Wang et al. (2019) demonstrated that knocking-down circRNA-ZFR inhibited cell proliferation, migration, invasion and induced apoptosis of CAKI-1 and ACHN cells; bioinformatics analysis data also showed that miR-206 was the candidate circRNA-ZFR target. In RCC cell lines, reduction of circRNA-ZFR significantly increased miR-206 expression; luciferase reporter assay results also verified circRNA-ZFR could directly sponge miR-206, and the decline of cell proliferation, migration and invasion capacity was alleviated by downregulating miR-206. As showed in this study, Met (met proto-oncogene) expression was markedly raised by reducing miR-206; previous studies had reported that Wnt/*β*-catenin and PI3K/AKT pathways were the downstream effectors of Met; both signaling pathways were in manage of multiple biological processes and frequently aberrantly activated in human cancers (Shorning et al., 2020; Zhang and Wang 2020). To determine the potential mechanism of circRNA-ZFR in RCC, western blot was carried out to detect the associated proteins expression. When circRNA-ZFR was silenced, the expression of Met, Wnt3a, *β*-catenin, PI3K and AKT was significantly reduced. Conversely, the expression of Met, Wnt3a, *β*-catenin, PI3K and AKT was significantly increased in CAKI-1 cells by decreasing miR-206. The above data suggested that circRNA-ZFR can promote RCC development by targeting the miR-206/Met axis to activate the Wnt/β-catenin and PI3K/AKT pathways.

## Circular RNA Zinc Finger RNA Binding Protein in Bladder Cancer

Luo et al. (2021) found that the expression of circRNA-ZFR was significantly higher in the tumor group than in the control group by analyzing the expression of circRNA-ZFR in 60 pairs of bladder cancer (BC) tissues and para-cancerous tissues, and circRNA-ZFR expression levels were positively correlated with tumor volume, TNM stage, and the proportion of metastatic lymph nodes. Immediately, circRNA-ZFR was shown to promote BC cell proliferation, migration and invasion both *in vivo* and *in vitro*; and as shown in the bioinformatics analysis, circRNA-ZFR was predicted to possess binding sites for miR-1270 and miR-545. The western blot and qRT-PCR detection results indicated that miR-1270 and miR-545 expression was significantly elevated in both T24 and J82 cells when circRNA-ZFR was attenuated. The results from RNA pull-down and dual-luciferase reporter assay also proved that circRNA-ZFR could directly interact with miR-545 and miR-1270. Otherwise, the probability of WNT5A (Wnt family member 5A) binding with miR-545 and miR-1270 was predicted by starBase, and these targeting relationships were verified by luciferase reporter assay. Previous studies had found that Wnt family genes played a vital part in human organogenesis and tumor genesis through regulating WNT/*β*-catenin signaling stimulate (Zhan et al., 2017; Krishnamurthy and Kurzrock 2018; Steinhart and Angers 2018). In addition, the expression of significant signaling components of the Wnt/*β*-Catenin pathway (PCNA, Ki-67, MMP-9 and N-catenin proteins) was significantly reduced by the attenuation of circRNA-ZFR, which was partially rescued by the application of miR-1270/545 inhibitors or WNT5A overexpression. Interestingly, Zhang H.,et al. (2019) found circRNA-ZFR was remarkably upregulated in tumor tissues and cell lines and related with poor prognosis of tumor patients. They also provided comprehensive evidences that knocking-down of circRNA-ZFR could effectively inhibit cell proliferation and migration by targeting the miR-377/ZEB2 axis, suggesting a potential therapeutic target for circRNA-ZFR in BC treatment.

## Circular RNA Zinc Finger RNA Binding Protein in Cervical Cancer

CC (cervical cancer) is one of the most common gynecological cancers in women all over the world. In the early stages, HPV-associated CC development is asymptomatic and its high invasiveness and mortality threaten the health of more and more women (Crosbie et al., 2013; Gaffney et al., 2018; Olusola et al., 2019). In the process looking for new biomarkers and intervention targets for the treatment of cervical cancer, Zhou et al. (2021) found that circRNA-ZFR high expression consistently could be identified as the biomarker for patients’ poor prognosis in cervical cancer. Especially, circRNA-ZFR overexpressed cells (HeLa and SiHa cells) were significantly exhibit a more malignant phenotype than control cells; cell cycle detection analysis revealed that the proportion of cells in S phase was significantly decreased after inhibiting the circRNA-ZFR expression, and a large number of cells were blocked in G0/G1 phase. By using Co-IP assay, it was found that circRNA-ZFR overexpression promoted the formation of CDK2/Rb, CDK2/Cyclin E1, and CDK2/SSBP1 complexes; conversely, attenuating circRNA-ZFR inhibited the formation of the same complexes. Subsequently, these complexes were proved to activate Rb/E2F1 pathway by inducting p-Rb S807 and S608 phosphorylation and activating E2F1 signaling. Interestingly, the Rb/E2F1 pathway activating could promote cervical cancer progression by allowing the transcription of genes required for the G1-S phase transition and DNA replication. In conclusion, circRNA-ZFR can act as a molecular scaffold by directly interacting with Rb to recruit CDK2, further promote the formation of related protein complexes and thus activate the Rb/E2F1 pathway. CircRNA-ZFR, a novel positive regulator of E2F1 signaling, could be a potential biomarker for cervical cancer detection.

## Circular RNA Zinc Finger RNA Binding Protein in Non-Small Cell Lung Cancer

By comparing the circRNA-ZFR expression between NSCLC (non-small cell lung cancer) tumor tissues and matching nontumor tissues of 45 patients, Tan et al. (2020) proved that there was a significant upregulation in the expression of circRNA-ZFR in NSCLC. And the analysis about the relationship between the relative circRNA-ZFR expression and clinical characteristics in patients with NSCLC indicated that circRNA-ZFR expression was an independent risk factor for cancer recurrence and poor prognosis in non-small-cell lung cancer. The cellular experiment results showed that circRNA-ZFR was also abnormally highly expressed in tumor cells; after exogenous silencing the circRNA-ZFR expression in tumor cells, the ability of cell proliferation, invasion and migration were decreased significantly. Due to the circRNA-ZFR downregulation, the percentage of NSCLC cells in G1 phase was significantly increased, indicating that more cells were blocked in the transition phase of G1/S phase. The bioinformatics tools prediction data suggested that miR-377-5p was a downstream target of circRNA-ZFR and NOVA2 (NOVA alternative splicing regulator 2) could also bind to miR-377-5p in NSCLC cells. Furthermore, the dual-luciferase reporter and RIP assays results were consistent with the concept that circRNA-ZFR could regulate NOVA2 expression by sponging miR-377-5p. The flow cytometry, wound healing and transwell invasion assay results demonstrated that circRNA-ZFR acted as an oncogenic molecule through sponging miR-377-5p in NSCLC cells. While, NOVA2 overexpression partially overturned miR-377-5p-mediated inhibition on the malignant potential of NSCLC cells. Collectively, circRNA-ZFR accelerated the proliferation, migration and invasion of NSCLC cells through miR-377-5p/NOVA2 axis.

Besides, the study of Zhang S. et al. (2019) showed that circRNA-ZFR was upregulated in NSCLC tissues and cell lines. CircRNA-ZFR knocking-down in NSCLC cells significantly inhibited cell proliferation, migration and invasion. According to the predicted results from bioinformatics tools, circRNA-ZFR might regulate CUL4B (cullin 4B) expression by directly targeting miR-101-3p; then luciferase reporter and RIP assays results validated these predictions. The miR-101-3p inhibitor transfection partially abrogated the inhibitory effect of circRNA-ZFR on NSCLC cells, and the CUL4B overexpression diminished the tumor suppressive effect of miR-101-3p on NSCLC cells. These results proved that circRNA-ZFR exhibited a carcinogenic role by sponging miR-101-3p to regulate CUL4B expression in NSCLC.

Currently, the treatment strategies for NSCLC include surgery, radiotherapy, chemotherapy and immunotherapy. Drug resistance in patients with NSCLC in the context of chemotherapy remains a major barrier to the treatment of NSCLC (Jonna and Subramaniam 2019; Proto et al., 2019). CircRNA-ZFR was found to be highly expressed in PTX-resistant NSCLC tissues and cell lines by Li et al. (2021). After exogenous restraint of circRNA-ZFR expression, the PTX-resistant NSCLC cells’ proliferation, migration and invasion abilities were significantly decreased; and flow cytometry data results exhibited circRNA-ZFR knockdown promoted cell cycle arrest and induced apoptosis in PTX-resistant tumor cells. As shown in starBase, miR-195-5p possessed the binding sites of circRNA-ZFR; and the inhibition of miR-195-5p ameliorated the effects of circRNA-ZFR knockdown on PTX sensitivity and cell progression in PTX-resistant NSCLC cells. In addition, KPNA4 (karyopherin subunit alpha 4) was found to be a target gene of miR-195-5p, dual-luciferase reporter assay and western blot results confirmed that miR-195-5p negatively modulated KPNA4 expression by direct interaction. The flow cytometry and transwell analysis results showed that miR-195-5p overexpression promoted apoptosis and inhibited cell migration and invasion in A549/PTX and H460/PTX cells, and this effect was preserved by increasing KPNA4. Subsequently, circRNA-ZFR silencing was found to significantly reduce KPNA4 mRNA and protein levels in A549/PTX and H460/PTX cells, while miR-195-5p inhibition effectively restored these effects. In conclusion, these results suggest that circRNA-ZFR promotes cell cycle progression, proliferation, migration and invasion, inhibits apoptosis and enhances patient’s PTX resistance in NSCLC by regulating miR-195-5p/KPNA4 axis. Similar to the above studies’ results, Li et al. (2020) revealed the involvement of circRNA ZFR as a molecular sponge in the regulatory mechanism of cisplatin resistance in NSCLC tumor cells by competitively sponging miR-545-3p and then inhibiting the binding of miR-545-3p to the downstream target gene CBLL1 (Cbl proto-oncogene like 1). CircRNA-ZFR participated in the negative regulation of miR-545-3p, which induced the isochronous expression with CBLL1 thereby achieving enhancing cisplatin resistance of NSCLC tumor cells *in vivo* and *in vitro*.

## Circular RNA Zinc Finger RNA Binding Protein in Esophageal Squamous Cell Carcinoma

Fang et al. (2020) confirmed circRNA ZFR expression was significantly higher in esophageal squamous cell carcinoma (ESCC) tumor tissue than in paraneoplastic tissue; qRT-PCR detection results also revealed that the significantly elevated circRNA-ZFR expression in different ESCC cell lines compared to the normal esophageal epithelial cells. The analysis results of the correlation between circRNA-ZFR expression and patients’ clinicopathological characteristics demonstrated that circRNA-ZFR overexpression was associated with the ESCC malignant phenotypes. In addition, circRNA-ZFR knocking-down inhibited the malignant biological behavior of ESCC cells, including proliferation, colony formation, migration, and invasion. The dual-luciferase reporter, RIP and qRT-PCR assay results suggested circRNA-ZFR could act as a sponge to absorb miR-377 in ESCC cells. VEGF (vascular endothelial growth factor), the most important regulatory factor in angiogenesis, had been proved to promote tumor growth and metastasis (Itatani et al., 2018; Apte et al., 2019; Peng et al., 2019). The prediction data suggested that VEGF is one of the potential binding targets of miR-377. Western blot and qRT-PCR results demonstrated that the miR-377 downregulation in ECA109 cells could significantly increase VEGF mRNA and protein expression levels; and the miR-377 overexpression could relatively reverse the up-regulated effects of circRNA-ZFR on VEGF protein. Taken together, circRNA-ZFR could regulate the VEGF expression to promote proliferation, migration, and invasion of ESCC by acting as a sponge for miR-377.

## Circular RNA Zinc Finger RNA Binding Protein in Hepatocellular Carcinoma

Xu et al. (2021) found that circRNA-ZFR level was obviously higher in HCC (hepatocellular carcinoma) tissues than that in normal tissues by qRT-PCR detection. Next, the relationship between the of circRNA-ZFR relative expression and clinical characteristics in patients with HCC was analyzed; the data results proved that high circRNA-ZFR expression was significantly associated with tumor node metastasis stage, tumor size and hepatitis B virus (HBV) infection, but not with patients’ age and gender. The functional experiments results indicated that the circRNA-ZFR down-regulation significantly decreased glucose uptake, lactate production and intracellular ATP content of Huh7 cells. Moreover, cell proliferation, migration and invasion were remarkably weakened due to the circRNA-ZFR down-regulation. The results of dual-luciferase reporter and RIP assays verified that circRNA-ZFR could directly target to miR-375; the silence of circRNA-ZFR inhibited the HCC cells progression by upregulating miR-375. As an important regulatory molecule of glucose metabolism (Zhang W. Y. et al., 2019; Unachukwu et al., 2020; Wu et al., 2020), HMGA2 (high mobility group AT-hook 2) was proved to be a downstream target gene of miR-375 involved in the regulation of HCC progression. The miR-375 overexpression or circRNA-ZFR reducing suppressed the HCC progression by downregulating HMGA2. The rescue experiments results showed that the inhibitory effects of silencing circRNA-ZFR and transfecting miR-375 mimics on glucose uptake and lactate production of Huh7 cells could be partially reversed by HMGA2 overexpression. In conclusion, this research demonstrated that circRNA-ZFR restraint suppressed glycolysis and proliferation of HCC cells via miR-375/HMGA2 axis.

As an important component of MAP kinase signal transduction pathway, MAP2K1 (mitogen-activated protein kinase kinase 1) has been proved to be involved in the regulation of cell proliferation, differentiation and gene transcription (Smits et al., 2020; Zhou W. Y. et al., 2020; Bu et al., 2021). Cedric’ study (2020) demonstrated that circRNA-ZFR was highly expressed in HCC tumor tissues and cells, correlated with the poor prognosis of HCC patients, and circRNA-ZFR was also proved to promote tumor cells’ proliferative capacity by targeting MAP2K1. Furthermore, Tan et al. (2019) and Yang et al. (2019) respectively revealed that abnormally high expression of circRNA-ZFR was closely associated with poor prognosis in patients with HCC. In the study of Tan, circRNA-ZFR was confirmed to accelerate HCC progression through regulating miR-3619-5p/CTNNB1 axis and activating Wnt/*β*-catenin pathway. Whereas, Yang et al. (2019) emphasized AKT1 (serine/threonine kinase 1) is a crucial receptor for activation of highly oncogenic Wnt/*β*-catenin signal pathway, which is closely linked to HCC cell proliferation and migration. The results of Yang’s research implied that circRNA-ZFR and AKT1 expressions were up-regulated and miR-511 expression was down-regulated in hepatocellular carcinoma. What’s more, circRNA-ZFR silencing or miR-511 overexpression suppressed cell proliferation, migration and invasion, and induced apoptosis of HCC cells. Mechanistically, circRNA-ZFR acted as a miR-511 sponge to up-regulate its target gene AKT1, and activated cascades of proliferation-related proteins (c-Myczd, cyclin D1, Survivin and Bcl-2). These data indicated that circRNA-ZFR might promote cell proliferation and migration by regulating miR-511/AKT1 axis in hepatocellular carcinoma.

## Circular RNA Zinc Finger RNA Binding Protein Exceptional Low Expression

The qRT-PCR detection results showed that circ-ZFR was drastically downregulated in gastric cancer tissues and cells. As confirmed by CCK-8 assay, overexpression of circ-ZFR observably suppressed GC (gastric cancer) cell proliferation; flow cytometry analysis disclosed that the apoptosis rate of GC cells was significantly increased after circRNA-ZFR overexpression, and the proportion of cells staying in G1/G0 phase significantly increased. Through starBase, a bioinformatics prediction website, Liu et al. (2018) found that there was a binding site between miR-107/miR-130a with circRNA-ZFR; and then, they crossed the targeting gene regulated by both p53 pathway and miR-130a/miR-107 *via* venn diagram, and the intersection was PTEN (phosphatase and tensin homolog). Moreover, The RIP and dual-luciferase reporter assay results indicated that circRNA-ZFR could target miR-107/miR-130a and PTEN was the downstream target gene of miR-107/miR-130a. The CCK-8 assay and flow cytometry analysis results displayed that circ-ZFR influenced GC cell propagation, cell cycle and apoptosis resistance by miR-107/miR-130a/PTEN axis. Interestingly, the rescue experiment results showed PTEN overexpression also attenuated the effects of circRNA-ZFR silence in GC cells, and the xenograft mice model experiment results showed that circRNA-ZFR curbed GC tumor growth and affected p53 protein expression *in vivo*. In general, circRNA-ZFR inhibited cell proliferation and promoted apoptosis in GC by miR-130a/miR-107/PTEN axis. By analyzing the circRNA-ZFR expression in 30 colorectal cancer tissues and matched non-tumor normal tissue specimens, Bian et al. (2018) confirmed that circRNA-ZFR expression was dysregulated in CRC tissues. In addition, they analyzed the effect of circRNA-ZFR on cell migration using wound healing and transwell migration assays, CCK8 and clone formation assays were also conducted to analyze cell proliferation; these results suggested that circRNA-ZFR restraint could promote the proliferation and migration of colorectal cancer cells. Bioinformatic techniques suggested that circRNA-ZFR and FOXO4 (forkhead box O4) share a common site of action on miR-532-3p; declining miR-532-3p by using siRNAs could increase the expression of circRNA-ZFR and FOXO4 in SW620 cells. Moreover, knocking-down miR-532-3p also promoted cell proliferation and migration. The western blot results showed reducing miR-532-3p could promote FOXO4 expression and mitigating circRNA-ZFR could reverse this phenomenon. All these results indicated that the circRNA-ZFR expression was down-regulated in gastric cancer and colorectal cancer, and circRNA-ZFR might play as a tumor suppressor in these cancers through the endogenous competitive mechanism.

## Conclusion and Perspective

With the research development, the aberrant expression of circRNAs has been gradually recognized as a hallmark feature in cancer. Investigating these molecules as biomarkers or therapeutic targets will be a promising field for cancer treatment (Liu et al., 2019; Pandey et al., 2020; Di Timoteo et al., 2020). Numerous studies have confirmed that circRNA-ZFR played very important roles in the occurrence and development of various tumors. CircRNA-ZFR was abnormally highly expressed in most malignant tumors such as bladder cancer and hepatocellular carcinoma, and promoted the malignant biological behaviors through numerous complex regulatory pathways; it could also act as a tumor suppressor in gastric cancer and colorectal cancer, and has a certain inhibitory effect on tumor proliferation, invasion and migration. Moreover, in non-small cell lung cancer, the abnormal expression of circRNA ZFR has a certain impact on patients’ chemotherapy sensitivity. Mechanistically, circRNA-ZFR could act as a competitive endogenous RNA in most tumors, exerting corresponding oncogenic effects by adsorbing miRNAs and thus regulating the expression of downstream target genes. The downstream miRNAs of circRNA-ZFR mainly include the miR-377 family (adsorbing miR-377 in bladder and esophageal squamous cell carcinoma and targeting miR-377-5p in non-small cell lung cancer), the miR-545 family (adsorbing miR-545 in bladder cancer and targeting miR-545-3p in non-small cell lung cancer) and other miRNAs. Furthermore, in cervical cancer, circRNA-ZFR could also act as scaffolding RNA to promote the formation of protein complexes by targeting CDK2 with RB and further regulating downstream signaling pathways. And the latter finding proved that there might be two or even more mechanisms of action of circRNA-ZFR in tumors, which might be related to the heterogeneity among tumors or the characteristics of tumors themselves, providing us with new ideas for the subsequent study of circRNA-ZFR. It is believed that with the deepening of research, the regulatory mechanisms of circRNA-ZFR in human malignant tumors will be more clearly displayed, which will provide new ideas and targets for tumor diagnosis and clinical treatment.
